# Key factors influencing magnetic nanoparticle-based photothermal therapy: physicochemical properties, irradiation power, and particle concentration *in vitro*†

**DOI:** 10.1039/d4na00384e

**Published:** 2024-11-12

**Authors:** Yilian Fernández-Afonso, Laura Asín, Juan Pardo, Raluca M. Fratila, Sabino Veintemillas, M. Puerto Morales, Lucía Gutiérrez

**Affiliations:** a Instituto de Nanociencia y Materiales de Aragón (INMA), CSIC-Universidad de Zaragoza Zaragoza Spain lu@unizar.es; b CIBER-BBN Zaragoza Spain; c Institute for Biocomputation and Physics of Complex Systems (BIFI), University of Zaragoza 50018 Zaragoza Spain; d Instituto de Ciencia de Materiales de Madrid (ICMM/CSIC) Madrid Spain

## Abstract

A collection of magnetic nanoparticles with average particle sizes in the range between 9 and 78 nm were prepared using several synthetic approaches that also provided different particle morphologies (spherical, octahedral and flowers). Some of these particles were also subsequently coated with different molecules in order to generate a set of materials that allowed us to evaluate the impact that the particle size, shape and coating had on the heating capacity of the nanoparticles when exposed to near infrared (NIR) laser light. Moreover, one of the prepared materials (octahedral particles of ∼32 nm coated with dextran) was used to perform an *in vitro* assay to study the possible use of this material in the frame of photothermal treatments to trigger cell death. It was found that both the laser power and the particle concentration played a significant role in the reduction of the cell viability. Under the most extreme conditions of laser power and nanoparticle concentration, cell viability was reduced to 11% of the whole cell population using only 10 min exposure to laser light. These results open the possibility of further studies of photothermal treatments using magnetic nanoparticles, a material already approved for clinical practice.

## Abstract 2

The photothermal properties of a collection of magnetic nanoparticles were analyzed. One of these sets of particles was further tested *in vitro* triggering cell death after 10 minutes of laser exposure.

## Graphical abstract

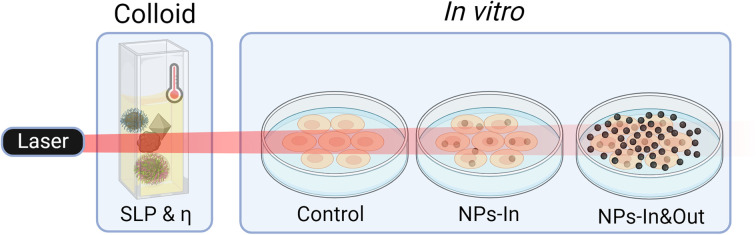

## 1 Introduction

The photothermal properties of iron oxide nanoparticles have become of interest in recent years.^1,2^ Although their heating properties may not be as strong as those of gold nanoparticles, with surface plasmon resonance absorption, the low toxicity of iron may make them interesting competitors in the frame of photothermal therapies.^3^ In addition, iron oxides have shown interesting synergistic heating effects when exposed both to NIR light and an AC magnetic field.^4^

One of the main problems for the knowledge advancement in this area is the existing difficulties to compare results obtained from different laboratories. A huge variety of experimental conditions are used to characterize the heating properties of the particles when exposed to near infrared (NIR) light, from different wavelengths to different volumes irradiated, among others.^5^ This plethora of measurement conditions hinders the comparison of results obtained from different laboratories using different materials.

The role of the different materials properties (size, shape, coating, aggregation and composition) in their heating properties under exposure to NIR light has been explored by different groups studying series of materials with small differences, obtaining sometimes strikingly different results. In 2015, Shen *et al.* reported the stronger heating capacity of multi-core nanoparticles forming nanoflowers in comparison with single core ones,^6^ increasing the interest in this type of clustered material. The next year, Guo *et al.* prepared a series of multicore particles with a size range between 60 and 310 nm, finding no significant differences in their heating properties due to the different final particle size.^7^ However, very recently, nanoflowers with particle sizes between 40 and 160 nm have been characterized by Shaw *et al.*^8^ showing an effect of the particle size on their heating properties, with the smaller particle sizes being the ones that produced stronger heat. In summary, the impact on clustering and the size of multi-core particles on their heating properties under exposure to NIR light remains difficult to predict.

The role of the particle coating in the heating performance of the material is difficult to disentangle from other properties and therefore is not fully understood yet. As an example, in 2014, Sadat *et al.* compared single-core particles, coated with PAA (poly(acrylic acid)), with the same particles but embedded in polystyrene spheres forming multicore structures either coated or uncoated with a silica film, reporting that the individual particles had a much higher photothermal heating efficiency than the clustered ones.^9^ Nemec *et al.* also recently reported a decrease in the heating properties of clustered particles in silica matrices when compared with their individual counterparts.^10^ However, in such types of studies, in which both the coating and the clustering are varied, it is difficult to extract which parameter is making a bigger effect on the heating properties of the materials.

Regarding their composition, most of the magnetic materials used for biomedical applications are generally iron oxides, mainly magnetite and maghemite (Fe_3_O_4_ and γ-Fe_2_O_3_). Recently, Roca *et al.*^1^ simulated the optical properties of both materials and found that Fe_3_O_4_ has better properties than γ-Fe_2_O_3_ for photothermal applications. However, as these two materials have an inverse spinel structure, it is often detected that both phases coexist within the iron oxide particles.^11^ Moreover, the addition of other metals to the ferrite structure would also have a significant effect on the heating properties of the particles. Rivero *et al.*^12^ prepared a series of zinc doped iron oxide ferrites with similar sizes but variable zinc content in the structure, finding that increasing the amount of doping agent reduced the heating efficiency of the particles. Nevertheless, the addition of other metals to the iron oxide structure has not been fully explored yet.

As a result, given that several parameters (size of the multi-core particles, interparticle distances, coating material, crystalline structure…) may play an important role in the heating properties of the material, drawing conclusions from the comparison of results provided by different laboratories becomes challenging. Therefore, further studies are needed to disentangle the impact of all these parameters on the heating properties of the materials.

Comparing *in vitro* literature results is also a complicated task. A profound review study by Roca *et al.* concluded that given the varied conditions across studies, such as different cell lines, nanoparticle concentrations, capping layers, laser parameters (power, spot size, and wavelength), and irradiation durations, it was difficult to draw general conclusions about the optimal therapeutic conditions.^1^ It is interesting here to mention that most of the *in vitro* studies until now have been performed using irradiation light with wavelenghts between 650 and 950 nm, in the first biological window of NIR light (NIR-I)^1,4,7,13,14^ and there are few *in vitro* studies in the second biological window (NIR-II, wavelength between 1000 and 1350 nm),^1,5,15,16^ where the maximum permissible exposure (MPE), meaning the light that can be applied without damaging the irradiated area, that can be achieved is much higher, allowing reaching higher temperatures.

In this work, in order to evaluate the impact that different particle properties have on the heating capacity of the material under exposure to NIR laser light, we have selected and characterized 12 different nanoparticles. This extensive catalog of iron oxide magnetic nanoparticles has been prepared to isolate different particle properties for their analysis of the impact on their heating properties. For example, to evaluate the impact of the particle size, spherical particles with different sizes but the same coating have been synthesized. Moreover, uncoated octahedral particles of different sizes have also been prepared. In addition, to evaluate the effect of the coating, particles with the same iron oxide core and different coatings have also been prepared. Finally, to evaluate the impact of aggregation, multi-core structures of different core sizes or particle sizes have been prepared. The heating capacity of the particles has been systematically measured. Finally, to go one step forward, one of these particles has been selected to perform a proof-of-concept *in vitro* study, to evaluate the capacity of the particles to trigger cell death. To achieve this, two cell models have been studied, one in which the particles were only located inside the cells (NPs-In), and other in which the particles were both inside the cell and in the extracellular media (NPs-In&Out). The second model, mimics better the real situation that may occur *in vivo* after an intratumor administration.

## 2 Experimental

### 2.1 Nanoparticle synthesis

Several synthetic approaches were used to cover a wide range of particle sizes and shapes.

Octahedral nanoparticles were prepared by the oxidative precipitation method^17–19^ with some modifications made to the method described by Andres *et al.*^20^ An FeSO_4_ (1 M) solution was prepared dissolving 13.90 g of FeSO_4_·7H_2_O in 50 mL of H_2_SO_4_ (0.01 M). The ferrous solution was quickly added to a basic solution prepared with 4.25 g of NaNO_3_ and 4.22 g of NaOH in a mixture of water and ethanol. The obtained iron(ii) hydroxide suspension was stirred for 15 min and placed at 90 °C for 24 hours. The whole process was carried out in a glove box in a nitrogen atmosphere. The final precipitate was washed with distilled water using magnetic separation. In water, the particle size is controlled by the excess of OH, in such a way that larger excess leads to larger particles (50–75 nm). When the reaction is carried out in the presence of ethanol, smaller particles are obtained (25–30 nm). The nanoparticles obtained were subjected to acid treatment and then, some were coated under high-pressure homogenization conditions with dextran^21^ and poly(sodium salt of acrylic acid).^19^

Flower-shaped nanoparticles were synthesized by the polyol synthesis method^22,23^ using a microwave reactor. For this, 0.13 g of FeCl_3_·6H_2_O were added to 20 mL of ethylene glycol in a beaker and placed under magnetic stirring. Then, 2 g of PVP40 was added slowly followed by 0.39 g of NaAc·3H_2_O. When all reagents were completely dissolved, the mixture was transferred to the reaction vial and placed in the reactor. The reaction was carried out at 200 °C with stirring. Different particle sizes were obtained by varying the reaction time (90, 120, and 240 min). The nanoparticles were washed with Milli-Q water and ethanol by magnetic separation. Finally, the particles were resuspended in Milli-Q water.

Spherical nanoparticles of 9 and 16 nm were obtained by the thermal decomposition of an iron oleate precursor in 1-octadecene in the presence of oleic acid.^24^ The reaction was carried out in a nitrogen atmosphere. For this, 3.5 g of iron oleate were mixed with oleic acid previously dissolved in 39 mL of octadecene and the mixture was placed in a heating mantle with stirring. The system was kept tightly closed, refrigerated and water refluxed. Two temperature ramps were performed: for the smallest particles the temperature was increased at 2.6 °C min^−1^ up to 320 °C (NPs-9), and for the bigger particles, the temperature was increased at 3.1 °C min^−1^ up to 200 °C, then it was maintained for 2 hours at this temperature and finally it was increased at 2 °C min^−1^ up to 320 °C (NPs-17). The reaction was finished after 60 min from the start of the maturation process at 320 °C. In order to generate a hydrophilic surface, the surface of these nanoparticles was modified using an amphiphilic polymer (poly(maleic anhydride-*alt*-1-octadecene) (PMAO, MW 30–50 kDa) following previously described protocols,^25^ resulting in particles named NPs-9@PMAO and NPs-17@PMAO.

### 2.2 Nanoparticle characterization

The nanoparticle size and shape were determined through TEM observations. Samples were prepared by placing a drop of the diluted suspension onto a carbon-coated grid and allowing it to dry at room temperature. Images were acquired on a Tecnai G2 TEM (FEI) operated at 200 kV.

Optical absorbance characterization was performed using magnetic nanoparticle suspensions at 0.1 mg_Fe_ mL^−1^ in the wavelength range of 300–1200 nm. A volume of nanoparticle dilution (100 μL) was placed in a quartz cuvette (3 mm optical path) and the absorbance spectra were acquired on a UV-Vis-NIR spectrophotometer (Jasco V670). The absorbance of the suspension with 1 mg_Fe_ mL^−1^ needed to calculate the photothermal efficiency was estimated from the experimental data obtained at 0.1 mg_Fe_ mL^−1^, considering that there was a linear correlation between the absorbance and the concentration.

The heating capacity of all the sets of particles was measured using colloidal suspensions of 1 mL with an iron concentration of 1 mg_Fe_ mL^−1^. Additionally, a water sample was used as a control. The experimental set-up (see Fig. S1 in the ESI†) was designed to allow the stirring of the particle suspension during the laser exposure. The nanoparticles were placed in a quartz cuvette with 1 cm of optical path length (*L*) and 0.4 cm of width (*W*), with a Teflon-coated magnetic stir bar (5 mm × 2 mm) inside. The cuvette was placed on a magnetic stirrer. A volume fraction of the suspension in the cuvette was irradiated with a laser at *λ* = 1064 nm (Quantum Laser, mpc6000/Ventus 1064, maximum power 3 W). The laser irradiation power used was 1.22 W; however, the power of sample irradiation was 1.17 W due to the cuvette wall absorption. The laser irradiation power was measured with a potentiometer (Ophir 10A). The laser beam diameter was 2.2 mm, approximately, so that the irradiated sample volume was 0.04 mL. The distance between the cuvette and the laser source was 20 cm. The temperature increase was recorded during the first 100 s using a thermocouple.

The calculation of the Specific Loss Power (SLP) was performed using the corrected slope method. Different methods can be used to estimate the heating efficiency of these colloidal nanoparticle systems: Initial Slope Method (ISM), Box-Lucas Method (BLM), Corrected Slope Method (CSM), Incremental Analysis Method (INCAM) and decay method among others.^26,27^ In this work, the corrected slope method was used for the analysis of the SLP. The CSM corrects the estimates made with the ISM by considering linear losses in the initial range of the heating curve. For this, several linear adjustments were made at small time subintervals in the initial curve, and the SLP was estimated using the following equation,^26,28^1
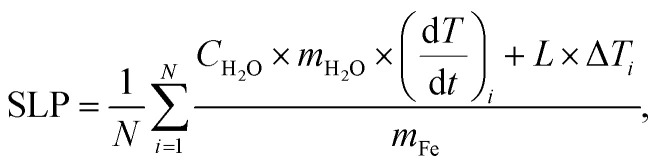
where *m*_H_2_O_ is the water mass corresponding to the total volume of the sample (1 mL), *m*_Fe_ is the iron mass of the sample volume irradiated (0.04 mL), (d*T*/d*t*)_*i*_ is the slope of the linear fit in the subinterval *i*, Δ*T*_*i*_ = (*T* − *T*_0_)_*i*_ is the mean value corresponding to the subinterval *i* and *L* is the thermal loss parameter and is estimated as the value giving the lowest standard deviation for the SLP values calculated in each subinterval *i*.

To calculate the SLP using de CSM, the initial heating curve was divided into four overlapping subintervals (0–16, 8–24, 16–32 and 24–40 s), a linear fit was performed at each subinterval and finally the SLP was calculated with eqn (1). The SLP was estimated from two measurements of each nanoparticle suspension.

### 2.3 *In vitro* test

The *in vitro* photothermal study was performed on murine macrophage cell line Raw 264.7. For this assay, cells were cultured and maintained in Dulbecco's Modified Eagle's Medium (DMEM) supplemented with 10% fetal bovine serum, 1% glutaMAX and 1% antibiotic (penicillin–streptomycin). Cells were seeded in a 96 well plate (10 000 cells in each well) and incubated during 24 hours to let the cells adhere. After that, 100 μL at 100 μg_Fe_ mL^−1^ (10 μg_Fe_) of NPs-32@DEX nanoparticles in DMEM were added to each well and incubated for 24 hours at 37 °C in a 5% CO_2_ atmosphere. Some of these wells were washed with DMEM to remove the particles that had not been taken up by the cells generating the NPs-In group. Other wells were kept without this last washing step, so all the particles remained within the well (NPs-In&Out group).

Cells in the well were irradiated with a NIR laser (Laser Quantum, mpc6000/Ventus 1064, maximum power 1.5 W) at 0.5 and 1 W. In order to expand the laser beam diameter and irradiate a larger area of the sample, a beam expander was placed between the source and the sample. The 96 well plate was placed on top of a surface with a hole (1 cm diameter) that only allows the laser to irradiate a single well. The laser was expanded to fit such a hole, irradiating an area just slightly larger than the well (0.28 cm^2^). The temperature of the sample during irradiation was acquired with an infrared camera (FLIR E4 Wifi). Cell morphology was analyzed before and after laser irradiation using an optical microscope (Nikon Eclipse TE2000-S).

Cell viability was studied through MTT (3-(4,5-dimethylthiazol-2-yl)-2,5-diphenyltetrazolium) assay 24 hours after being irradiated with a laser. The medium was removed and washed 2 times with 100 μL of Dulbecco's phosphate buffered saline (DPBS). A volume of 90 μL of DMEM and 10 μL of MTT (5 mg mL^−1^) was added and after 2 hours of incubation at 37 °C, purple formazan crystals formed and the wells were centrifuged at 2500×*g* for 25 minutes. After removal of the supernatant, 100 μL of dimethyl sulfoxide (DMSO was added to dissolve the crystals formed and finally the absorbance was measured at 550 nm on a Varian Cary 100 UV-Vis spectrophotometer. Each sample was analyzed in triplicate.

Cell viability was also analyzed by flow cytometry using an apoptosis kit (Alexa Fluor 488 – Annexin V/Dead cell (Invitrogen)). To detach the cells from the bottom of the wells 24 hours after being irradiated with the laser, these were washed with PBS, incubated with 50 μL trypsin and fresh DMEM was added. The cells were centrifuged, washed with PBS and resuspended in 100 μL of 1× annexin-binding buffer. After that, 5 μL Alexa Fluor 488 annexin V and 1 μL PI working solution (100 μg mL^−1^, previously prepared according to manufacturer's instructions) were added and incubated at room temperature for 15 minutes. A volume of 400 μL 1× annexin-binding buffer was added, mixed gently and the samples were kept on ice. Measurements were performed on a CytoFLEX flow cytometer (Beckman Coulter) and approximately 5000 cells were analyzed in each case and the results were processed using CytExpert software.

Control cells and cells incubated with the particles and washed (NPs-In) were subjected to nitric acid digestion (<1% of final volume) before elemental analysis to determine the Fe (corresponding to nanoparticles) uptake by cells. Iron concentration in the digested samples was measured using an Inductively Coupled Plasma Atomic Emission Spectrometer (ICP-AES) PerkinElmer model OPTIMA 2100 DV.

### 2.4 Statistical analysis

One-way ANOVA (*p* > 0.05 no significance) was used to compare the SLP and photothermal efficiency values from the different nanoparticles tested. Two-way ANOVA (*p* > 0.05 no significance) followed by a Bonferroni test to compare the means was used to compare the cell viability results of the three analyzed groups (Control, NPs-In and NPs-In&Out) obtained from the MTT assay.

## 3 Results

### 3.1 Preparation and characterization of a library of magnetic nanoparticles

**Fig. 1:**
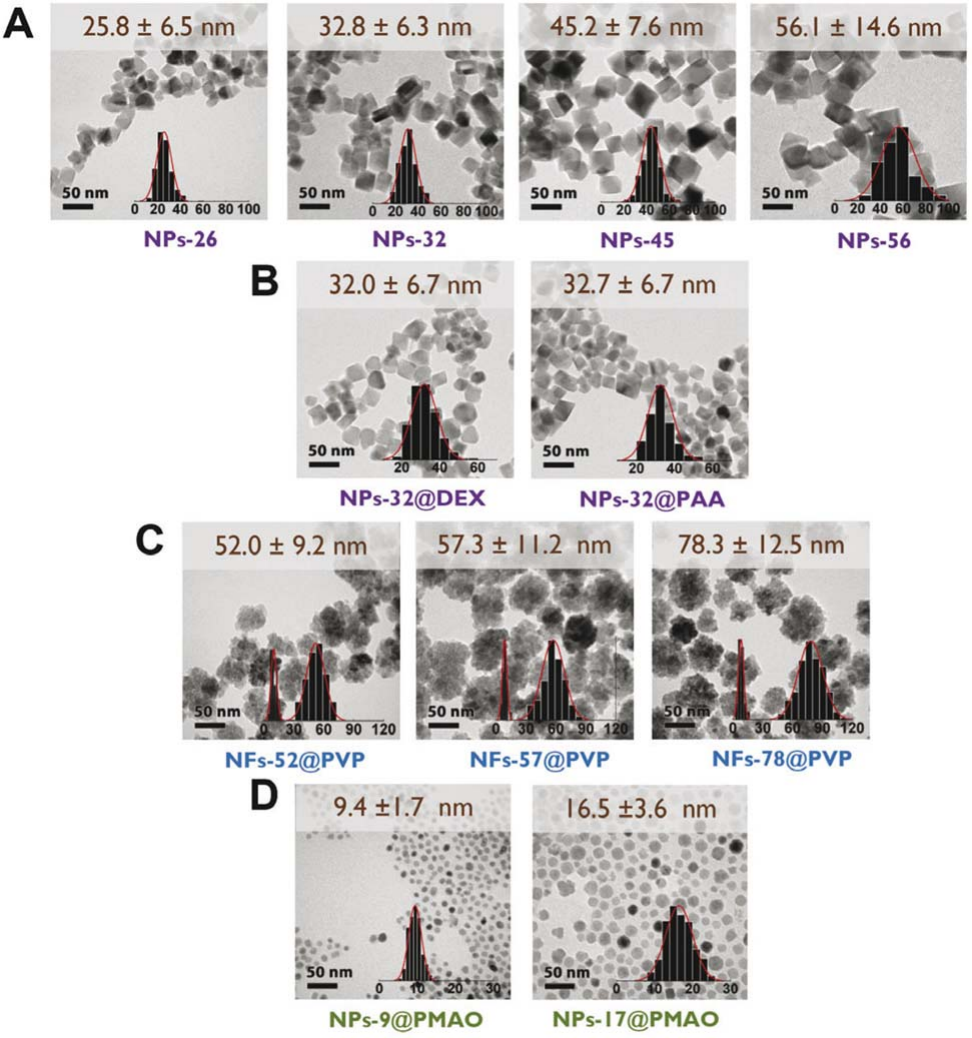
TEM images and particle size distribution: (group A) uncoated octahedral nanoparticles with sizes between 26 and 56 nm, (group B) 32 nm octahedral nanoparticles with different coatings, (group C) nanoflowers with the same core size (8–10 nm) and coating (polyvinylpyrrolidone) and different particle sizes (52–78 nm), and (group D) spherical nanoparticles with sizes of about 9 and 17 nm coated with PMAO.

Several synthetic approaches including oxidative precipitation, thermal decomposition and polyol synthesis were used to produce a complete library of iron oxide magnetic nanoparticles that included different types of particle size, coating and aggregation (Fig. 1). These procedures are known to produce either magnetite, maghemite or intermediate compositions with the oxidation of magnetite into maghemite,^29^ and in some case have been scaled up to grams.^17^

Octahedral particles (Fig. 1A) were synthesized following a protocol previously described^15^ in which an iron(ii) salt (FeSO_4_) is precipitated in the presence of a base (NaOH) and a mild oxidant KNO_3_. Variations of this procedure (see Experimental section) yielded four types of uncoated particles with sizes in the range between 26 and 56 nm (NPs-26, NPs-32, NPs-45 and NPs-56). The NPs-32 particles were also used to produce samples with the same core size but different coatings: NPs-32@DEX, coated with dextran, and NPs-32@PAA, coated with polyacrylic acid (Fig. 1B). All particles after synthesis and before coating were subjected to an acid treatment for improving colloidal properties and stability against oxidation with time. Coating was carried out by high-pressure homogenization leading to hydrodynamic sizes below 100 nm.

Multicore particles (Fig. 1C) were prepared using a polyol synthesis method and a microwave reactor. This synthetic route allowed the production of multicore particles (usually termed nanoflowers) in which both the core size and the nanoflower size could be modified. In this synthetic route, polyvinylpyrrolidone was used as part of the synthesis reagents and it remained as a particle coating. Using this synthetic procedure we were able to prepare particles in which we observed the same core size, but the nanoflower size was different (NFs-52-PVP, NFs-57-PVP and NFs-78-PVP, with a core size of ∼8–10 nm).

Spherical particles (Fig. 1D) were prepared by thermal decomposition of either iron acetylacetonate or iron oleate precursors in the presence of oleic acid following previously described protocols.^25,30^ This synthesis produces particles that are only stable in organic media and therefore a coating protocol using PMAO was used, to be able to transfer them to an aqueous medium.^25^ This approach allowed us to prepare smaller particles than with the previously described synthesis route. Two sets of particles of 9 and 16.5 nm average particle size were prepared (NPs-9@PMAO and NPs-17@PMAO).

**Fig. 2:**
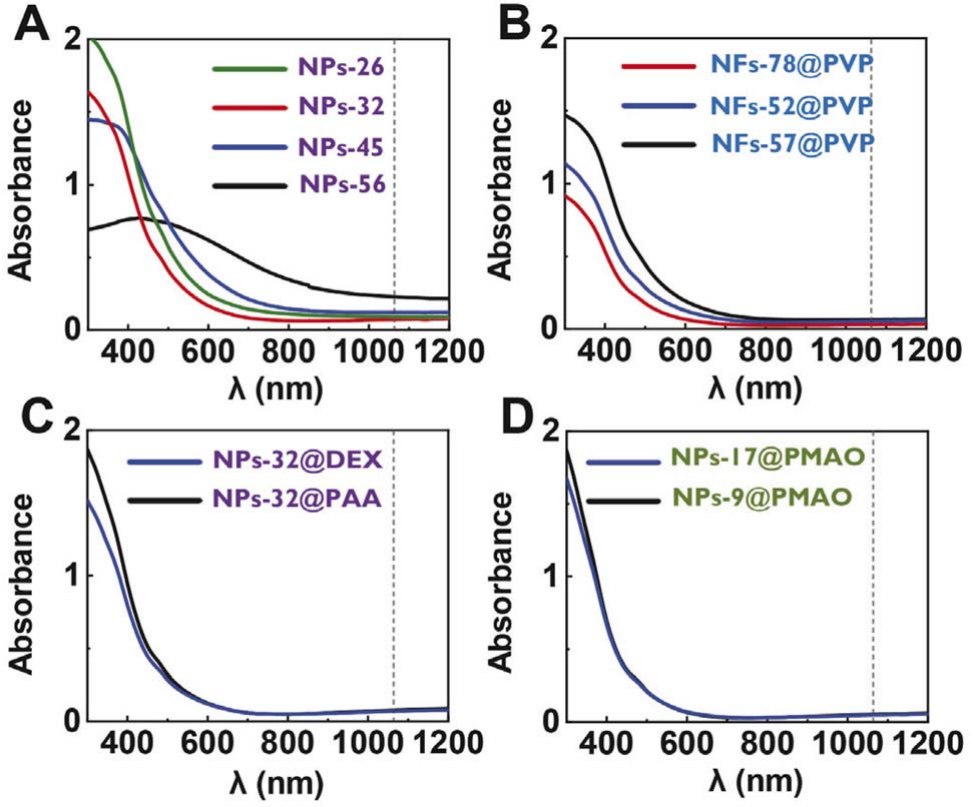
UV-Vis-NIR spectra at 0.1 mg_Fe_ mL^−1^ in the range of 300–1200 nm of (A) uncoated octahedral nanoparticles with sizes between 26 and 56 nm, (B) nanoflowers with the same core size (8–10 nm) and coating (polyvinylpyrrolidone) and different particle sizes (52–78 nm), (C) 32 nm octahedral nanoparticles with different coatings, and (D) spherical nanoparticles with sizes of about 9 and 17 nm coated with PMAO.

Optical absorbance is a parameter required for the photothermal efficiency calculations. Measurements of the absorbance of all the prepared particles were performed with colloidal suspensions with the same iron concentration in the wavelength range of 300–1200 nm. In general, the spectra showed a maximum at wavelengths below 450 nm, together with a decrease in the absorbance from the near-UV to the NIR region (Fig. 2). Although significant differences were found among the absorbance values of the particles at the lowest wavelengths, smaller differences were found for all particles at the wavelength of interest (1064 nm) independently of the particle size, shape or coating, with the absorbance values being in the range between 0.1 and 0.2. Additional differences that could occur at this wavelength, associated with the crystalline structure of the materials being either magnetite or maghemite,^1^ were also not observed here. In our case, previous studies on octahedral particles obtained through the same synthetic procedure have reported the coexistence of both magnetite and maghemite.^11^

For the subsequent analysis of the heating performance of the particles, we chose 1064 nm as the excitation wavelength. The reasons for this selection were that this particular wavelength is part of the second biological window, the good performance of iron oxides at such a wavelength, the higher maximum permissible exposure (MPE) at *λ* = 1064 nm and the lower number of studies using magnetic nanoparticles performed in NIR-II in comparison with the more widely studied first biological window.^1^

**Fig. 3:**
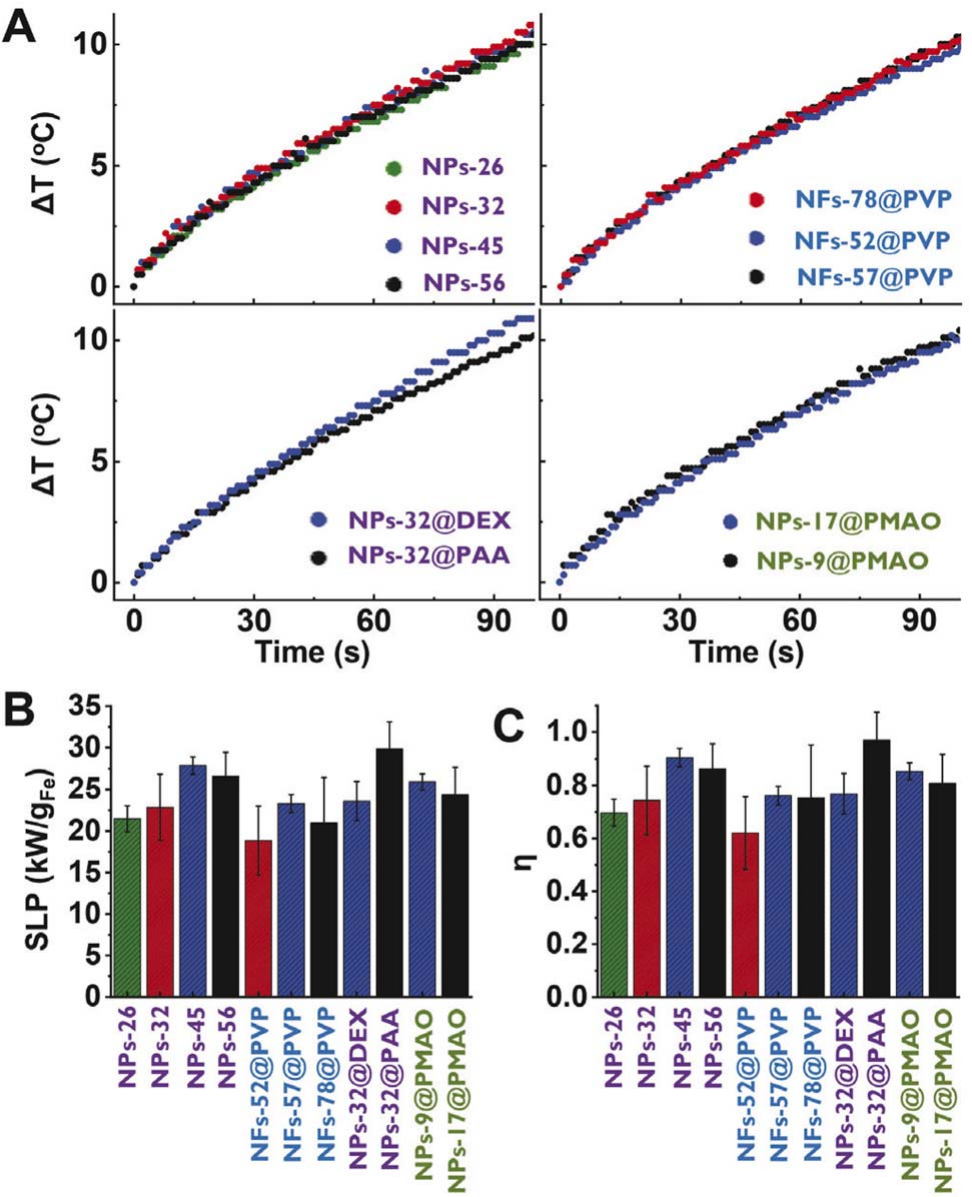
(A) Heating measurements of the different nanoparticle suspensions at 1 mg_Fe_ mL^−1^ (1 mL) using a 1064 nm laser and 1.17 W power. Two measurements for each particle suspension were performed. (B) SLP values of the nanoparticle suspensions calculated using the Corrected Slope Method (CSM). (C) Photothermal efficiencies of nanoparticle suspensions. Statistical significance between the means from data in panels (B) and (C) was determined using a one-way ANOVA (*p* > 0.05 no significance). No significantly different values were found.

The heating capacity of all the sets of particles was measured using colloidal suspensions of 1 mL with an iron concentration of 1 mg_Fe_ mL^−1^. The nanoparticles were placed in a quartz cuvette that was irradiated from one side. As not the whole particle suspension could be irradiated (the laser beam diameter was 2.2 mm), the experimental set-up was designed to allow the stirring of the particle suspension during the laser exposure (see the Experimental section) to allow the measurement of a homogeneous temperature. The temperature increase was recorded during the first 100 s using a thermocouple. Additionally, a water sample was used as a control, showing a very low temperature variation (Δ*T*_100s_ = 1.4 °C) over time (see Fig. S2 in the ESI†). All samples displayed very similar heating curves (Fig. 3A) regardless of the size, shape, or coating. In photothermal treatments, the heating produced by iron oxide particles is related to the amount of light that they can absorb.^1^ As in this case, absorbances determined at 1064 nm were very similar for all cases, it makes sense that similar heating curves were also obtained. Therefore, the differences in size, shape, aggregation or coating studied in this case did not have a great impact on the heating properties of the particles.

The heating efficiency of magnetic nanoparticle suspension is generally quantified through the Specific Absorption Rate (SAR) or the Specific Loss Power (SLP). Although several methods have been proposed to estimate the heating efficiency of these colloidal nanoparticle systems (Initial Slope Method (ISM), Box-Lucas Method (BLM), Corrected Slope Method (CSM), Incremental Analysis Method (INCAM), decay method, *etc.*),^26,27^ all of them present some limitations for their application. The ISM and INCAM models consider that the system is adiabatic and therefore heat losses are not considered. The CSM, BLM and decay method consider that heat losses have a linear behavior; however, linear losses can only be considered in a limited temperature range. As described in the Experimental section with more details, in this work, the corrected slope method was used for the analysis of the SLP. At first sight, the average SLP value for each particle presented great variability (Fig. 3B), despite the similarity between heating curves. However, the statistical analysis revealed that values calculated for all the particles were not significantly different. This uncertainty in the determination of the SLP values may be one of the reasons why so many different behaviors are observed when comparing results described in the literature. SLP values are very difficult to compare between different authors in the literature because they depend a lot on measurement setups and irradiation conditions.

Thus, in addition to SLP, the photothermal efficiency of the samples was determined. The photothermal efficiency is an intrinsic property of the sample and depends mainly on the size, shape, composition and coating of the nanoparticle and solvent. Therefore, the photothermal efficiency values obtained using different configurations and different irradiation conditions can be directly compared as long as similar materials are characterized.

The photothermal efficiency (*η*) of all nanoparticles was calculated using eqn (2) (ref. 3)2
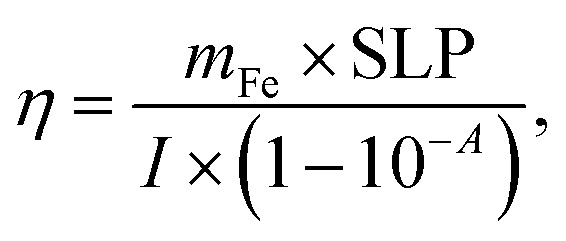
where *m*_Fe_ is the irradiated iron mass, *I* (1.17 W) is the laser power incident on the sample and *A* is the absorbance of the sample at the irradiation wavelength.

As the SLP is needed to calculate the photothermal efficiency, the variability observed in the SLP values in our case was thus translated into the photothermal efficiency ones (Fig. 3C). Again, the statistical analysis revealed that values were not significantly different. Moreover, no specific trends were observed for those series of particles in which just one parameter (either the size, shape or coating) was varied. Strikingly, a wide range of photothermal efficiency values was determined for all particle suspensions (0.5 < *η* < 1).

As explained in the introduction, contradictory results regarding the effect that different physicochemical properties have on the heating performance of magnetic nanoparticles for photothermal treatments have been previously reported. A similar behavior to what we observed in this work was reported by Sadat *et al.*^9^ In their work, similar heating curves were reported for 10 nm PAA-coated nanoparticles and agglomerates of these nanoparticles embedded in a matrix of polystyrene that in some cases was further coated with silica. In contrast, Zhao *et al.* characterized similar samples and found differences in their heating curves.^31^ In addition, other studies had also showed some differences in heating curves between uncoated and silica-coated 12 nm particles and 95 nm agglomerates.^10^ Moreover, previously reported photothermal efficiency values of iron oxide nanoparticles using different experimental and calculation approaches have shown a great disparity of values. For example, lower photothermal efficiency values have been reported: 0.29 for iron oxide nanocubes (20 nm) irradiated at 808 nm^3^ and 0.21 for aggregated nanoparticles (200 nm) irradiated at 1064 nm.^16^ Other studies have also reported values in the same range as our results: 0.76 photothermal efficiency for nanoparticles (10 nm) irradiated at 808 nm.^9^

Despite the contradictory results found in the literature, our results indicate the weaker sensitivity of the photothermal performance of iron oxide particles to size changes, and this behaviour may be especially relevant for further *in vivo* applications in which nanoparticles will degrade over time.^32^

### 3.2 *In vitro* test of the photothermal activity of the particles

In order to go one step forward and test the real activity of these particles in a photothermal treatment, a murine macrophage cell line (Raw 264.7) was selected for the *in vitro* test given the high capacity of this cell line to take up nanoparticles observed in previous studies.^33–35^ The NPs-32@DEX particles were selected for this study. These particles were selected given that iron oxide nanoparticles coated with dextran have already been approved by the FDA (U.S. Food and Drug Administration) for clinical applications.^36^

**Fig. 4:**
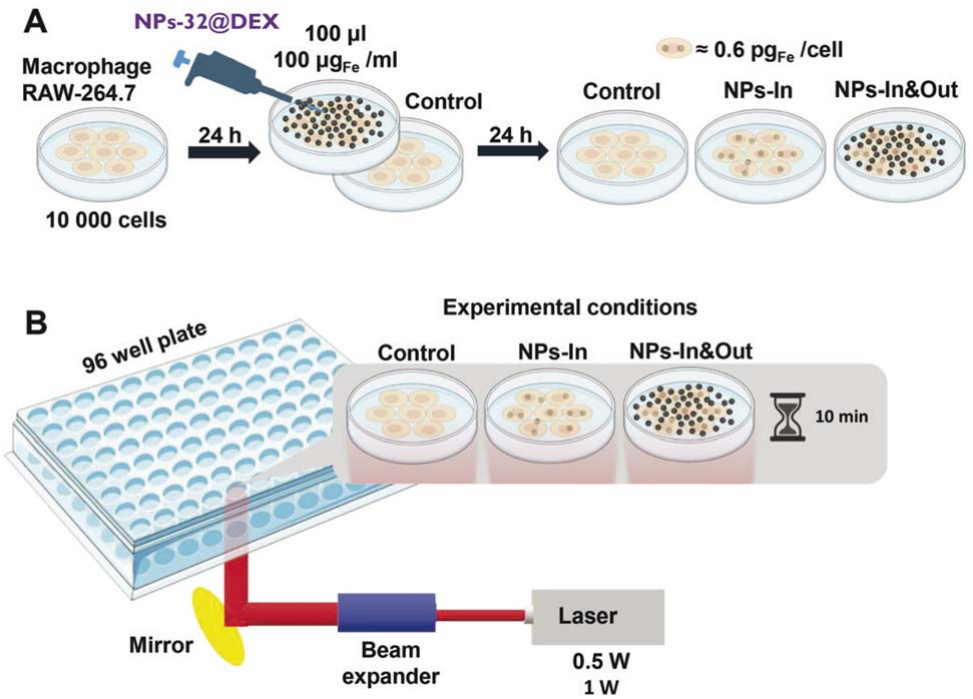
Schematic representation of the *in vitro* tests showing: (A) the three types of cell treatment: (i) the control group, which was not incubated with NPs, (ii) the NPs-In group consisted of cells incubated with the particles in which the particles that were not taken up were subsequently washed, so there were no particles in the cell culture media, and (iii) the NPs-In&Out group, in which the cell culture medium was not removed and particles were located both inside and outside the cells; and (B) scheme of the irradiation procedure. Figure created with https://www.Biorender.com.

Cells were seeded in a 96 well plate and 100 μL of the NPs-32@DEX suspension with an iron concentration of 100 μg_Fe_ mL^−1^, equivalent to a total mass of iron of 10 μg_Fe_, was added to each well. Cells were incubated with the particles for 24 hours and after that, two different strategies were used: either the cells did not receive any additional treatment so all the particles were kept within the well (NPs-In&Out) or they were washed to remove the particles that had not been taken up by the cells (NPs-In) (Fig. 4A).

Using the same laser as described before, several changes in the set-up were made to allow the irradiation of individual wells from the bottom. A holder for the 96 well plate with a specific-sized hole was used, allowing irradiation of a single well (Fig. 4B). Additionally, a laser beam expander was employed to fit the diameter of the hole and enable irradiation of the entire well.

**Fig. 5:**
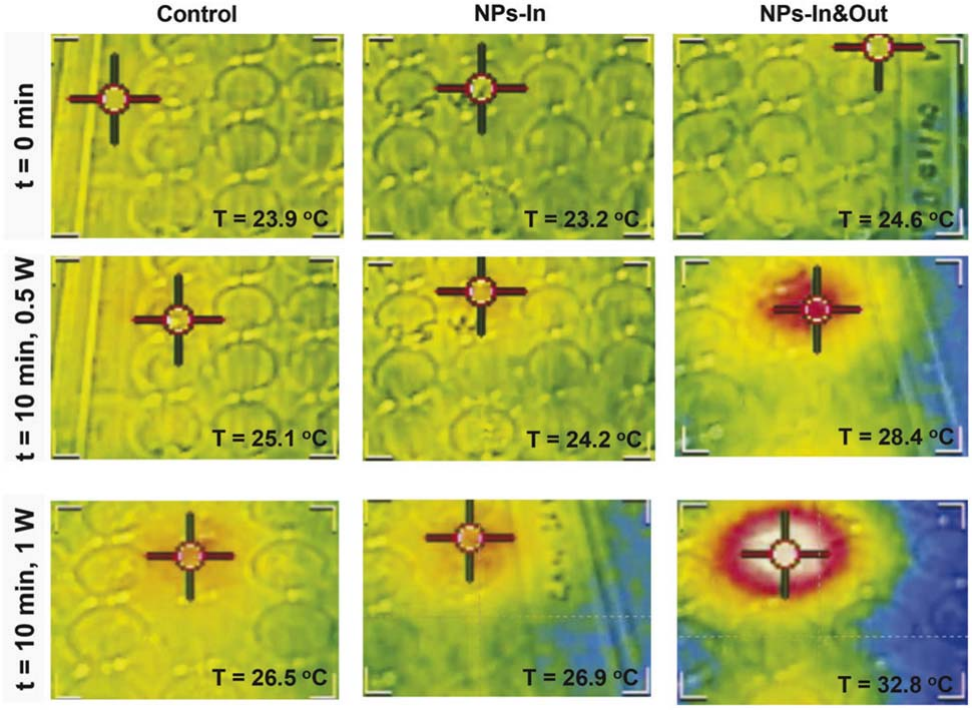
Representative images, taken with an infrared camera, of the well plate in which the cells were treated with the laser. The two upper rows correspond to different time points of the experiment at the same laser power (0.5 W), the top row is the initial time point while the bottom row corresponds to the same well at the final time point (10 min of NIR irradiation). The lower row corresponds to the final time point of an experiment performed at a higher laser power (1 W). Each of the columns corresponds to a different group of cells: (left) control cells, (middle) NPs-In and (right) NPs-In&Out. The color scale is related to the temperature going from blue in the coldest area to white in the hotter one (blue < green < yellow < red < white). The temperature indicated in the image corresponds to that on the irradiated well.

An infrared camera was used to record the temperature of the irradiated wells (Fig. 5). An increase in temperature of ∼1 °C was observed over a 10 min period both for the control cells and the NPs-In group when irradiated with 0.5 W laser power. A slightly higher temperature increase was achieved in these two groups when the power was increased to 1 W (∼2.5 and ∼4 °C for the control and NPs-In group). Interestingly, a remarkable difference was observed for the NPs-In&Out group compared to the other two groups when exposed to both 0.5 and 1 W laser powers. For the NPs-In&Out group, an increase of the global temperature of ∼4 °C was detected when using the 0.5 W power and an ∼8 °C increase was measured using the 1 W power, both within the same time frame. This observation is clearly related to the larger amount of NPs present in this group.

In the case of the NPs-In&Out sample, the total amount of iron administered to each well was considered (10 μg_Fe_, that accounts both for particles inside and outside the cells). However, it was necessary to measure the amount of iron in the control cells and cells incubated with the particles and washed (NPs-In). ICP-AES measurements revealed values of iron mass per well several orders of magnitude different for the two groups with particles. In contrast with the 10 μg_Fe_ for the NPs-In&Out, only 19 ng_Fe_ were measured for the NPs-In, which corresponds to 0.6 pg_Fe_ per cell that would mainly be located within the lysosomes.^37,38^ These values clearly explain the different temperatures reached in both groups during the treatment.

**Fig. 6:**
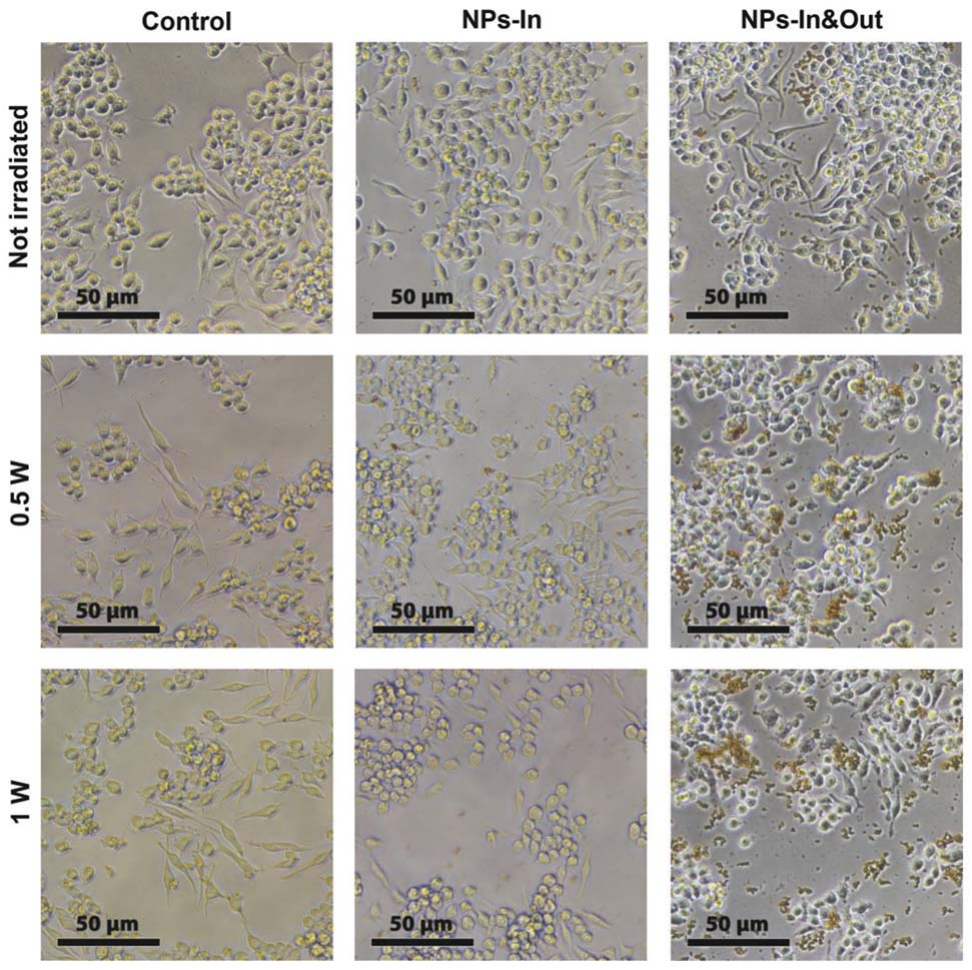
Optical microscopy images of the cells taken before (top row) and 24 h after the photothermal treatment (bottom rows). Each of the columns corresponds to a different group of cells: (left) control cells, (middle) NPs-In and (right) NPs-In&Out group.

**Fig. 7:**
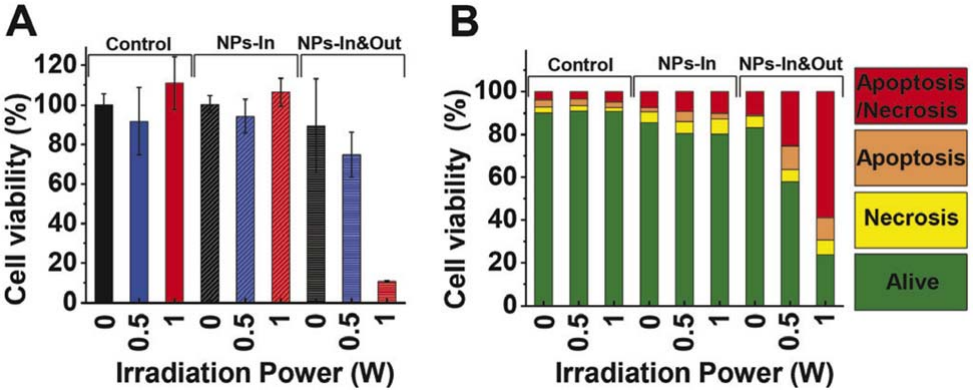
Cell viability study after irradiation during 10 min using two laser powers (0.5 and 1 W). Percentage of cell viability analyzed by (A) MTT assay and (B) flow cytometry for control, NPs-In, NPs-In&Out groups. Dot blots are shown in Fig. S3 in the ESI.†

The morphology of the cells was assessed by optical microscopy before and after the irradiation. While no significant differences were found in the control group and the NPs-In group, fewer cells were found in the wells corresponding to the NPs-In&Out group 24 h after the treatment and also some changes in morphology were observed for those remaining cells. Under normal conditions, RAW cells typically present a heterogeneous morphology, some of them being more rounded and others more spread like, which makes this cell line to be not the most convenient one to study the effect of any stimulus in terms of morphology change. However, the cells in the NPs-In&Out group 24 h after the irradiation showed a slight loss of adherence and spread morphology, with most of them, compared to the other conditions, floating on the cell medium. This observation was an indication that cell death could be triggered under these conditions both at 0.5 and 1 W of laser power (Fig. 6). In order to quantify the cell death and elucidate the mechanism triggered after the photothermal treatment, cell viability was studied 24 h after the irradiation using two different viability tests: a cell proliferation test (MTT assay) and an apoptosis-necrosis assay for flow cytometry (Fig. 7).

The MTT assay showed that neither the MNPs nor the irradiation alone produces cell damage in terms of a cell viability decrease. Besides, the NIR laser treatment in the group NPs-In does not induce any alteration in the cell viability 24 hours post treatment. However, cell viability decreased in the NPs-In&Out group 24 h after the irradiation during 10 min (Fig. 7A). Indeed, a cell viability of 75% was observed in the group irradiated with 0.5 W power. A further decrease of the cell viability (down to 11%) was observed in the same group but irradiated with 1 W laser power.

This assay measures the activity of mitochondrial enzymes that reduce the MTT (3-(4,5-dimethylthiazol-2-yl)-2,5-diphenyltetrazolium bromide) to a purple formazan product. The information that this assay gives is a combination of the mitochondrial metabolic state of the cells and its proliferation capability, but it does not provide any information regarding the type of cell death occurring. So, in order to complement these results, an apoptosis-necrosis assay by flow cytometry was performed.

Two different biological events were studied and analyzed by flow cytometry for elucidating the cell death mechanism triggered by the treatment. First, the cell membrane permeability was studied by using propidium iodide (PI) that penetrates the cells when the membrane is damaged (either during late apoptosis or necrosis). Besides, the translocation of the phosphatidylserine (PS) was evaluated. PS is a molecule normally expressed in the internal part of the plasmatic membrane but translocated to the outer part of the membrane in the initial stages of apoptosis. Cells were incubated with annexin V that binds the PS. The analysis of these two markers allowed the identification of cells that were alive (negative for both markers), cells in an early apoptosis stage that present translocation of PS maintaining the membrane integrity (positive only for annexin V), necrotic cells that presented membrane integrity alteration (positive only for PI) and those in a late apoptosis or late necrosis stage (positive for both annexin V and PI).

Flow cytometry results showed that the cell viability remained above 80% for the control and NPs-In groups after the irradiation either with 0.5 or 1 W of irradiation power (Fig. 7B). As was also observed in the MTT assay, 24 h after the treatment cell viability was significantly reduced for the NPs-In&Out group achieving a cell death rate of 42% and 76% for the two irradiation powers. In both cases, most of the dead cells were in a late apoptosis/late necrosis stage. Similar results were found by Cabana *et al.* showing also that 24 h after the irradiation most of the dead cells were positive for both PI and annexin V, indicating that cells undergo a similar death mechanism to the one observed with our nanoparticles and our experimental conditions.^39^ Although shorter analysis times would be needed to confirm which cell death mechanisms were occurring, a higher proportion of cells was found in an early apoptosis stage, compared to the necrosis one, so probably the irradiation is causing cell death through apoptosis.

Here, it is important to mention that although most of the studies using photothermal treatments have focused on the generation of an increase in temperature as a cause of cell death (*e.g.*, though the analysis of the production of heat shock proteins^40^), recent studies have also pointed out the photothermal irradiation accelerates nanoparticle degradation inside cancer cells leading to Fe^2+^ release, ROS generation, lipid peroxidation and cell death through ferroptosis.^41^

Our results indicate that even if the particles administered do not achieve enough internalization to generate a great cell death (NPs-In model) *per se*, having the particles also in the extracellular environment (NPs-In&Out model) is helpful to reduce cell viability after photothermal treatments. This result is especially interesting, as in a real application, if the particles are administered intratumorally, the NPs-In&Out model better mimics the clinical scenario^42–45^

Related to this, the work of Cabana *et al.*^39^ showed that when comparing cell viability on a PC3 prostatic cancer cell line using two types of iron oxide particles after photothermal treatment, cell death was only generated with the biggest particles. However, those biggest particles were also the ones showing much larger internalization. In that work, the authors stated that the smaller particles were then not suitable for therapy under such conditions. Although it is difficult to compare our results with others in the literature, as generally several parameters (nanoparticle size, amount of NPs taken up, cell type, irradiation conditions, *etc.*) differ among the different studies, our results, showing the effect of the extracellular particles on cell death would open the possibility to use particles that are not well internalized for the therapeutic treatment.

Indeed, a recent study by Lázaro *et al.*^46^ evaluated the effect of the cell uptake on the cell death after the photothermal treatment. They found that the location of the particles was fundamental to ensure the cytotoxic effect of photothermal treatments, showing that lower NP concentration was needed if those particles were located intracellularly instead of extracellularly. However, it is important to highlight that in an *in vivo* scenario, probably a combination of particles located inside and outside the cell will occur, similar to our NPs-In&Out model.

In summary, our findings suggest that magnetic nanoparticles, even if not efficiently internalized by cells, hold potential for photothermal treatments, expanding the range of materials that could be used for this biomedical application.

## 4 Conclusions

A set of 12 different iron oxide magnetic nanoparticles was prepared to analyze the impact of the particle size, shape and coating on their heating capacity when exposed to NIR laser light in the frame of photothermal therapies. No significant differences were observed between the different materials, both in the SLP and the photothermal efficiency. These results point to the need of a standardized methodology to characterize the heating properties of these materials in order to be able to compare literature results.

Furthermore, one of the characterized particles (NPs-32@DEX) was selected to perform *in vitro* tests to evaluate the efficacy of the photothermal treatment in order to generate cell death. Cells were incubated with the particles and two different models were prepared. The NPs-In model was generated by removing all the NPs that were not taken up by the cells after the incubation, and leaving only those particles inside the cells. In contrast, the NPs-In&Out model consisted of cells incubated with the particles in which the cell medium was not removed, so in addition to the uptaken NPs, there were also particles within the cell culture medium.

These two models were irradiated during 10 min with two different laser powers (0.5 and 1 W). A remarkable global temperature increase was detected for the NPs-In&Out model compared to the other groups. This difference was associated with the higher NP concentration in such groups of cells.

Moreover, cell viability was assessed by different methods (optical microscopy images, MTT assay and flow cytometry). The results from all these techniques were in agreement showing a much higher decrease of the cell viability in the NPs-In&Out group than the NPs-In group. Moreover, within the NPs-In&Out group, a higher decrease of the cell viability was observed when the irradiation power was 1 W compared to 0.5 W, reaching cell viabilities between 11 and 24%, depending on the technique used for the analysis, for the 1 W power.

These results indicate that magnetic nanoparticles are able to trigger cell death within photothermal treatments, but to modulate the biological outcome both the laser power and the amount of particles need to be optimized to generate the required cell death.

## Data availability

All data presented in this work, including TEM images and origin files with data used to generate the figures will be uploaded at Zenodo repository.

## Author contributions

Yilian Fernández-Afonso: conceptualization; formal analysis; investigation; validation; visualization; writing – original draft, Laura Asín: conceptualization; formal analysis; investigation; validation; writing – original draft, Juan Pardo: investigation; methodology, Raluca M. Fratila: funding acquisition; investigation; writing-review and editing, Sabino Veintemillas-Verdaguer: investigation; methodology, M. Puerto Morales: funding acquisition; investigation; writing-review and editing and Lucía Gutiérrez: conceptualization; funding acquisition; methodology; project administration; supervision; visualization; writing – original draft.

## Conflicts of interest

The authors declare no conflicts of interest.

## Supplementary Material

NA-007-D4NA00384E-s001
