# Supplementary material for: Key factors influencing magnetic nanoparticle-based photothermal therapy: physicochemical properties, irradiation power, and particle concentration in vitro
Source: Nanoscale Adv. 2024 Nov 12;7(1):336–45. doi: 10.1039/d4na00384e (PMC11609872; doi:10.1039/d4na00384e)
Supplement: NA-007-D4NA00384E-s001 [file NA-007-D4NA00384E-s001.pdf]

Supplementary Information (SI) for Nanoscale Advances.
This journal is © The Royal Society of Chemistry 2024

Key Factors Influencing Magnetic Nanoparticle-Based Photothermal Therapy: Physicochemical Properties, Irradiation Power, and Particle Concentration *In Vitro*

April 30, 2024

Yilian Fernández-Afonso, Laura Asín, Juan Pardo, Raluca M. Fratila, Sabino Veintemillas, M. Puerto Morales, Lucía Gutiérrez*

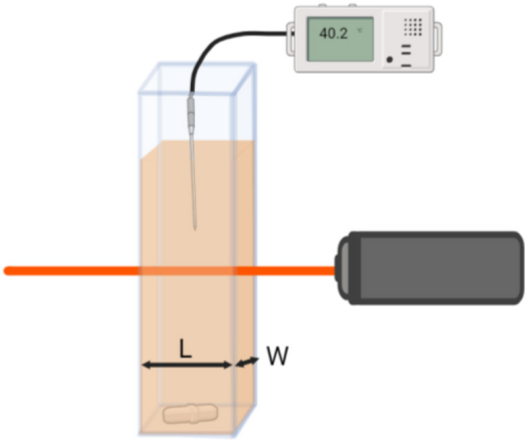

Figure 1: Experimental set-up scheme. Quartz cuvette with 1 cm of optical path length (L) and 0.4 cm of width (W), with a teflon-coated magnetic stir bar (5 mm x 2 mm). Laser irradiation wavelength was 1064 nm.

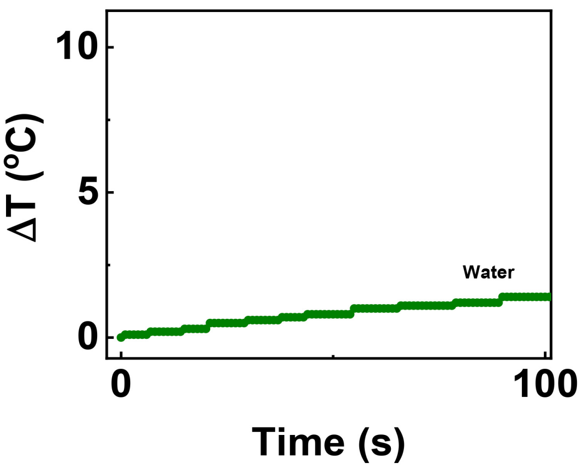

Figure 2: Heating measurement of a water sample using a 1064 nm laser and 1.17 W power.

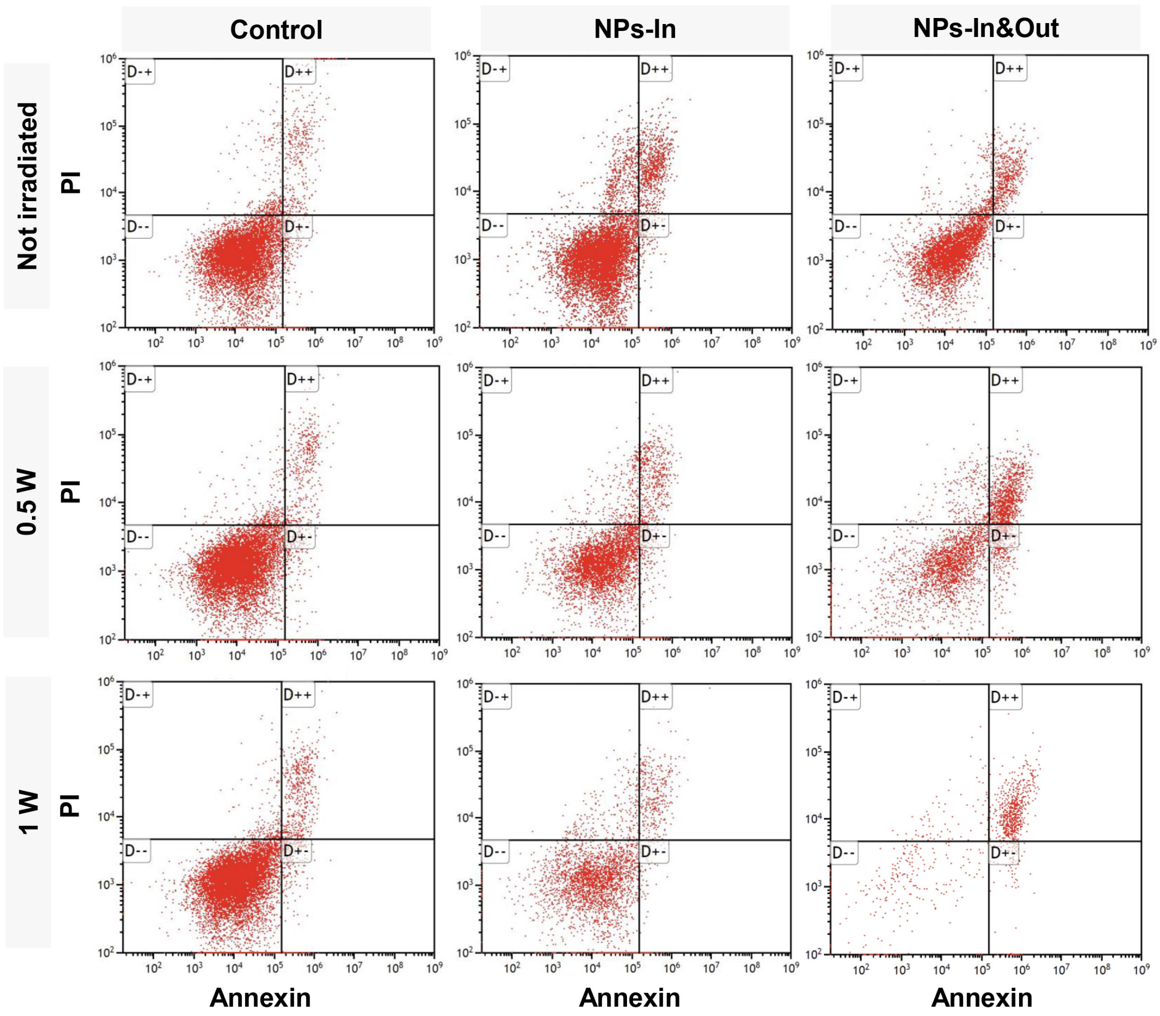

Figure 3: Flow cytometry studies. Dot blots corresponding to different analysed groups (Control, NPs-In, NPs-In&Out) irradiated with a 0.5 and 1 W power during 10 min.
